# Overexpression of PFKFB3 promotes cell glycolysis and proliferation in renal cell carcinoma

**DOI:** 10.1186/s12885-022-09183-2

**Published:** 2022-01-20

**Authors:** Jun Li, Shiqiang Zhang, Dingzhun Liao, Qian Zhang, Chujie Chen, Xiangwei Yang, Donggen Jiang, Jun Pang

**Affiliations:** 1 Department of Urology, Kidney and Urology Center, The Seventh Affiliated Hospital, Sun Yat-sen University, 628 Zhenyuan Road, Shenzhen, 518107 China; 2 Department of Pathology, The Seventh Affiliated Hospital, Sun Yat-sen University, Shenzhen, 518107 China; 3 Department of Rehabilitation Medicine, The Seventh Affiliated Hospital, Sun Yat-sen University, Shenzhen, 518107 China

**Keywords:** Renal cell carcinoma, PFKFB3, Glycolysis, Proliferation, G1/S transition

## Abstract

**Background:**

Cancer cells prefer utilizing aerobic glycolysis in order to exacerbate tumor mass and maintain un-regulated proliferative rates. As a key glycolytic activator, phosphofructo-2-kinase/fructose-2,6-bisphosphatase 3 (PFKFB3) has been implicated in multiple tumor type progression. However, the specific function and clinical significance of PFKFB3 in renal cell carcinoma (RCC) are yet not clarified. This investigation assessed PFKFB3 roles in RCC.

**Methods:**

PFKFB3 expression levels were analyzed in clear cell renal cell carcinoma (ccRCC) tissues, together with its relationship with clinical characteristics of ccRCC. Real-time PCR and Western blot assays were employed for determining PFKFB3 expression in different RCC cell lines. Furthermore, we determined the glycolytic activity by glucose uptake, lactate secretion assay and ECAR analysis. CCK-8 assay, clone formation, flow cytometry and EdU assessments were performed for monitoring tumor proliferative capacity and cell-cycle distribution. Furthermore, a murine xenograft model was employed for investigating the effect of PFKFB3 on tumor growth in vivo.

**Results:**

PFKFB3 was significantly up-regulated in RCC specimens and cell lines in comparison to normal control. Overexpression of PFKFB3 was directly correlated to later TNM stages, thus becoming a robust prognostic biomarker for ccRCC cases. Furthermore, PFKFB3 knockdown suppressed cell glycolysis, proliferative rate and cell-cycle G1/S conversion in RCC cells. Importantly, in vivo experiments confirmed that PFKFB3 knockdown delayed tumor growth derived from the ACHN cell line.

**Conclusions:**

Such results suggest that PFKFB3 is a key molecular player in RCC progression via mediating glycolysis / proliferation and provides a potential therapeutic target against RCC.

**Supplementary Information:**

The online version contains supplementary material available at 10.1186/s12885-022-09183-2.

## Background

The global prevalence for renal cell carcinoma (RCC) is on the rise, with over 400,000 novel RCC diagnoses and a mortality rate of over 170,000 in 2018 alone [1]. Clear cell renal cell carcinoma (ccRCC) is the most prevalent form of RCC (approximately 70–75% of all RCC cases) [2]. Radical / partial nephrectomy could possibly be the solution for early-stage, non-metastatic RCC, however, many cases present with late-stage RCC at the time of diagnosis since earlier stages are typically asymptomatic [3].

Bespoke therapeutic measures for vascular endothelial growth factor (VEGF), together with innovative immunotherapeutic options have been approved and used against late-stage / metastatic RCC. However, such therapeutic effects are transient and the patient eventually relapses [4]. Hence, it is essential to achieve enhanced knowledge on the molecular mechanisms for tumorigenesis and development in RCC in order for ensuring reliable guidance for rapidly identified therapeutic strategies. One potentially effective such emerging therapeutic option bases itself on exploiting the programming pathways for tumor cell metabolic processes, through the use of glycolysis inhibiting agents [5].

Exacerbated proliferative rates couple with de novo glucose-processing metabolic models are signature trends in tumors, in order to attain enough metabolic support to aid rapid tumor development through over-driven glycolysis, albeit having access to adequate oxygenation – this is known as the Warburg effect [6, 7]. Investigation of such intertwining mechanisms can provide further clues on tumorigenesis. During glycolysis, Phosphofructo-2-kinase/fructose-2,6-biophosphatase 3 (PFKFB3) leads to catalysis for fructose-2,6-bisphosphate (F-2,6-BP) production, a highly effective 6-phosphofructo-1-kinase 1 (PFK-1) allosteric inducer [8]. Consequently, PFKFB3 activating processes were correlated to exacerbation of glycolytic activity, with PFKFB3 also being up-regulated in multiple tumors including pancreatic [9], breast [10] and gastric cancer [11]. Up-regulated PFKFB3 levels demonstrated correlation with low survival statistics in breast cancer cases [12]. Interestingly, PFKFB3 is mainly localized in the nucleus, which is different from other members of the PFKFB family (PFKFB1–4). The unique localization might reveal the unexpected role of PFKFB3 in promoting cell proliferation through regulation of multiple, essential cell-cycle protein expression profiles, namely cyclin-dependent kinase-1 (CDK1) and p27 [13]. Gene silencing / chemically-driven impairment of PFKFB3 certainly applies molecular brakes on over-driving glycolysis activity, Ras-mediated transformative processes and also tumor mass increases in nude murine models [14, 15]. All such findings render PFKFB3 an important drug-target molecule in other cancer models. However, tumorigenic influences by PFKFB3 on RCC has been little explored.

This investigation focused on the evaluation of PFKFB3 expression profiling in 90 ccRCC patients and consequently assessed possible links between PFKFB3 expression and resulting clinical / pathological prognostic outcomes in such patients. Furthermore, this study investigated whether PFKFB3 knockdown inhibited glycolytic activity and regulates proliferative rates accordingly, both at in vitro and in vivo levels.

## Methods

### Patients and tissue samples

The tissue microarray (TMA) consisted of 90 pairs of ccRCC biopsies / matched juxta-tumor healthy biopsies, attained from Shanghai Outdo Biotech Company. All patients received no additional treatment before surgery. Patients were further divided into subgroups using the TNM stage classification: stage I (*n* = 55), II (*n* = 21), III (*n* = 8), IV (*n* = 6), and Fuhrman grade system: Grade I (*n* = 34), II (*n* = 39), III (*n* = 13), IV (*n* = 4). The investigation was carried out in full conformity with the Declaration of Helsinki and accepted by the Ethics Committee of the Affiliated Hospital of Xuzhou Medical University. All study participants handed informed-consent forms.

### Immunohistochemistry (IHC)

IHC assays were conducted as previously described [16], using anti-PFKFB3 [1:250, Abcam™, ab181861], and anti-Ki67 antibodies [1:250, Abcam™, ab15580]. Since PFKFB3 protein is primarily located in the nucleus, H-score was applied to assess the immunoreactivity of PFKFB3 according to the staining intensity and percentage. Cell stain scoring ranged from 0 to 3 (0 = no staining; 1 = mild; 2 = noticeable; 3 = extensive), where the scoring value was consequently multiplied by the percentage positive-staining cells (0% ~ 100%) in order to determine H-score (range = 0–300). Each slide was independently assessed and averaged by two pathologists without prior knowledge of patient data.

### Cell culturing

The *H. sapiens* RCC cell lines (ACHN, Caki-2, A498, 786-O and OS-RC-1) and a human renal proximal tubular epithelial cell line (HK-2) were obtained from the American Type Culture Collection (ATCC). Cells were cultured in RPMI-1640 [HyClone™, USA], together with 10% fetal bovine serum [Invitrogen™, USA] and 1% penicillin / streptomycin [HyClone™, USA] at 37 °C with 5% CO_2_.

### Establishment of stable cell lines with PFKFB3 knockdown

Bespoke lentiviral vectors carrying a sequence specific for PFKFB3 short hairpin RNA (sh PFKFB3) expression (5′-AGCCCGGATTACAAAGACTGCTTCAAGAGAGCAGTCTTTGTAATCCGGGCTTTTTTT-3′) or a scramble control sequence (5′-GCGCGATAGCGCTAATAATTTTTCAAGAGAAAATTATTAGCGCTATCGCGCTTTTTT-3′) were obtained from GenePharma™ [Shanghai, China]. RCC cells were transduced accordingly and selected with puromycin as previously described [17]. Finally, stable ACHN and A498 cells silenced for PFKFB3 expression were established and verified using Western blot.

### RNA isolation and real-time quantitative PCR

RNA was collected using TRIzol® [Invitrogen™, USA] and cDNA was produced using the RevertAid® First Strand cDNA Synthesis kit [Takara™, Japan]. Real-time PCR was carried out on ABI-7500, employing SYBR® Premix Ex Taq [Takara™, Japan]. β-actin served as the normalization control. Primer sets employed were: PFKFB3 forward 5′-GGCC GCATCGGGGGCGACTC-3′ and reverse 5′-TTGCGTCTCAGCTC AGGGAC-3′; β-actin forward 5′-GGGACCTGACTGACTAC-3′ and reverse 5′-TCATACTCCTGCTTGCTGAT-3′. All results were normalized against β-actin expression, with all fold changes determined through the 2^-ΔΔCT^ method.

### Western blot assay

Western blot analysis was conducted according to published protocols [16]. Cell lysis took place within RIPA buffering solution. All protein levels were normalized through the BCA® assay kit [Thermo Fisher Scientific™, USA]. β-actin served as loading control. Anti-PFKFB3 [1:1000, Abcam™, ab181861], anti-β-actin [1:3000, Abcam™, ab179467] and secondary antibodies labelled with HRP (1:3000, Abcam™, ab6747) were used in this study. The bands were visualized using ECL-plus western blotting detection reagents (BD Biosciences, USA).

### Glucose uptake and lactate secretion analysis

Glucose and lactate concentration was determined using a Glucose Colorimetric Assay Kit and a Lactate Assay Kit [BioVision™, USA], according to manufacturer instructions. Statistical differences were calculated compared with the negative control. Briefly, cells were seeded at a density of 2000 cells per well in a 96-well plate and incubated at 37 °C overnight. Cells were starved in serum free culture medium for 2 h. Cells were then washed with PBS and incubated with 100 μL Krebs-Ringer-Phosphate-HEPES (KRPH) buffer containing 2% BSA for 40 min for the depletion of endogenous glucose, followed by 10 μL 2-deoxyglucose (10 mM) incubation for 20 min. Cells were collected with extraction buffer and treated for detection of glucose uptake ability. The glucose uptake levels were measured by OD at 412 nm in a microplate reader and normalized to protein concentration. All the experiments were performed in triplicate.

For lactate secretion assay, cells were seeded at a density of 2000 cells per well in a 96-well plate and incubated at 37 °C overnight. After starvation for 2 h, the supernatant of each group was collected and cells were collected for extracting protein, following by measurement of lactate production. The lactate levels were measured at 450 nm in a microplate reader and normalized to protein concentration. All the experiments were performed in triplicate.

Protein assays were used to do normalization and were done according to Bicinchoninic Acid Protein Assay (Thermo Fisher Scientific, MA) protocol. Briefly, proteins were extracted from per well using RIPA lysis buffer (Beyotime Biotechnology), then protein reagent was added to another 96-well assay plate and mix with samples or standard, and then incubated at 37 °C for 30 min. The absorbance was read on a spectrophotometer at 562 nm.

### Extracellular acidification rate (ECAR) analysis

The glycolysis capacity was obtained through the Seahorse XF® Glycolysis Stress Test Kit [Agilent™, Santa Clara, USA], in line with manufacturer protocol. Briefly, cells were seeded at a density of 2000 cells in a 96-well plate and incubated overnight. After washing the cells with Seahorse buffer, 10 mmol/L glucose, 1 mmol/L oligomycin, and 100 mmol/L 2-deoxy-glucose (25 mL of each) were added to measure the ECAR. ECAR values were calculated and normalized to the cell number. The Agilent Seahorse system we used provides an XF data normalization solution using an automated imaging and cell counting workflow. In our experiments, we set the ECAR value per 10,000 cells for normalization, the Agilent Seahorse XF system automatically calculated and generated the normalized results.

### CCK-8 viability assay

Cell Counting Kit-8 (CCK-8) assay was used according to the manufacturer’s protocol. Cells transfected with PFKFB3 shRNA or treated with 10 μM 3PO (PFKFB3 inhibitor) were seeded in 96-well plates at a density of 2000 cells per well. Next, 10 μL of CCK8 solution (KeyGEN, Nanjing) were added to each well at 450 nm was measured 0, 1, 2, 3, and 4 days after seeding using a microplate reader (Spark 10 M, Shenyang, China). The experiments were carried out in triplicate.

### Clone formation assay

1000 cells were seeded in 6-well plates in the culture medium. After 10 days, the cells were fixed with 4% formaldehyde and visualized by crystal violet staining, clones were harvested when over 50 cells per clone were counted. The experiments were carried out in triplicate.

### Cell cycle analysis

For cell cycle analysis, cells were synchronized with serum deprivation and then released by serum re-addition. After staining with propidium iodide using Cycletest Plus DNA Reagent Kit (KeyGEN, Nanjing) according to the manufacturer’s protocol, the cell cycle distribution was analyzed using fluorescence-activated cell sorting (FACS) cytometry (Beckman Coulter™, USA). The percentages of cells in G0/G1, S and G2/M phases were counted and compared. The experiments were carried out in triplicate.

### EdU assay

Cells were seeded in 96-well plates (2000 cells/well) and treated with 100 μL of medium containing 20 μM EdU. After incubation for 2 h, the cells were fixed with 4% paraformaldehyde for 30 min and incubated with 0.5% Triton X-100 in PBS for 20 min. The nuclei were stained with Hoechst 33,342. The rate of proliferation was calculated following the manufacturer’s instruction (Ribo Biotech, Guangzhou). Images of five randomly selected areas of each group were taken with a fluorescence microscope (Leica, Germany).

### Small-molecule inhibitor of PFKFB3

The commercial PFKFB3 inhibitor, 3-(3-Pyridinyl)-1-(4-pyridinyl)-2-propen-1-one (3-PO) was purchased from Merck Millipore. Cells were cultured in the medium added 3PO (10 μM) as the experimental group, and the control group was absence of 3PO. After incubation for 24 h, glycolysis flux (glucose uptake and lactate secretion analysis) and cell viability (CCK8 assay) were detected. The experiments were carried out in triplicate.

### Tumor xenograft growth in the nude murine model

Murine model investigations were executed as described in previous protocols [18]. Twenty-eight-day-old male BALB/c nude murines were collected from Sun Yat-sen University Experimental Animal Center. 5 × 10^6^ ACHN cells (stably expressing sh Ctrl or sh PFKFB3) were subcutaneously injected into murine dorsal thighs (*n* = 6 murines per group). The tumor sizing was registered weekly, with tumor volume (mm^3^) being determined by:

$$\text{Volume}=0.5\times\text{length}\times\text{width}^2$$

Five weeks later, the murines were sacrificed through CO_2_ inhalation, followed by tumor nodule dissection, weighing, sectioning and IHC analysis intervention. The investigation was accepted by the Experimental Animal Care Commission of Sun Yat-sen University. Interventions conducted as part of this investigation were in full conformity with the ethics standard guidelines of the institutional ethics committee and with the NC3Rs ARRIVE guideline.

### Statistical analysis

The results were presented as the mean ± SD from a minimum of three independent runs. Statistical analysis was performed using SPSS 16.0 software-package. Any group variations were determined through Student’s t test or one-way ANOVA. Kaplan-Meier survival curve-based analyses were conducted and evaluated through the log-rank test. All statistical analyses were two-sided, with P < 0.05 being the threshold for statistical significance.

## Results

### PFKFB3 protein is overexpressed in human ccRCC specimens and positively correlates with late TNM stage and poor prognosis of ccRCC patients

**Fig. 1:**
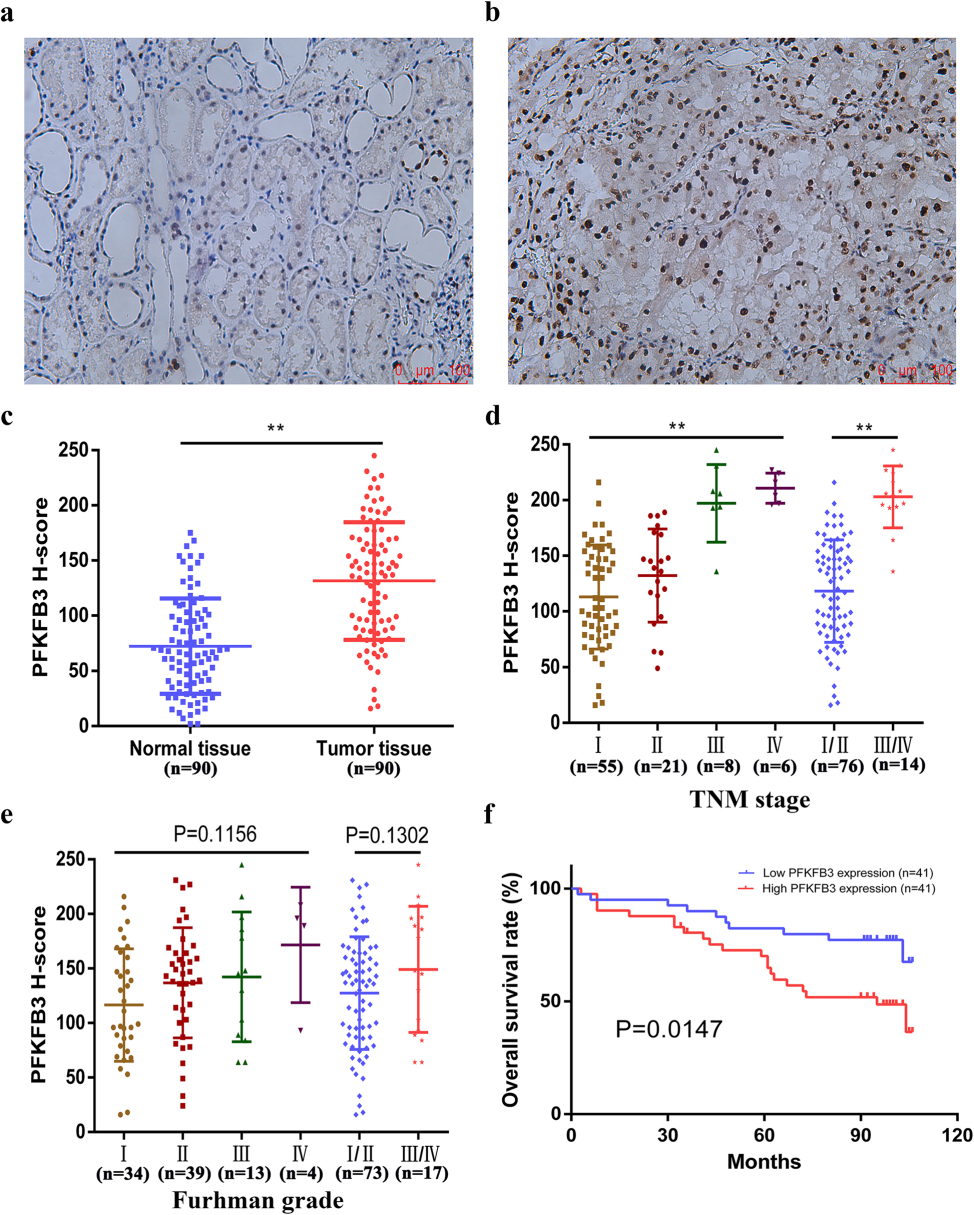
PFKFB3 protein is overexpressed in human ccRCC specimens and positively correlates with late TNM stage and low prognostic odds in ccRCC cases. (**a**) Representative images of juxta-tumoral healthy biopsies with down-regulated PFKFB3 expression. (**b**) ccRCC biopsy imaging, with high expression of PFKFB3. (**c**) H-score plot for PFKFB3 in ccRCC tissues (*n* = 90) and adjacent normal tissues (n = 90). (ccRCC = 131.52 ± 5.61 vs. Normal = 72.36 ± 4.55, P < 0.01). (**d-e**) H-score of PFKFB3 at multiple stages (TMN) and grades (Fuhrman) in ccRCC cases. (**f**) Kaplan-Meier overall survival times in ccRCC cases based on PFKFB3 expression (mean ± SD. *, P < 0.05; **, P < 0.01)

Using IHC, the PFKFB3 protein expression level in ccRCC mass and juxta-tumor healthy tissue was evaluated. As shown in Fig. 1a-b, this study demonstrated that PFKFB3-immunostaining signals were intense in the nucleus of ccRCC cells, though weak in healthy renal tissues. The H-score of the ccRCC tissues and juxta-tumor normal tissues were 131.52 ± 5.61 and 72.36 ± 4.55, respectively, while there were statistically significant variations among both tissue groups (Fig. 1c). Through analysis of the relationship between PFKFB3 expression level and pivotal clinical / pathological outcomes in ccRCC cases, this study elucidated that up-regulated PFKFB3 expression was present in late TNM stage (ANOVA P < 0.01, Stage I/II vs III/IV, P < 0.01, Fig. 1d). In addition, although PFKFB3 expression was up-regulated in high Fuhrman grade cases, this was not statistically significant (ANOVA *P* = 0.1156, Grade I/II vs III/IV, *P* = 0.1302, Fig. 1e). Additionally, this study explored the prognostic value of PFKFB3. Two patient groups were designated, depending on PFKFB3 expression levels, namely: low PFKFB3 (H-score<median, *n* = 41) and high PFKFB3 (H-score>median, n = 41). Eight patients were excluded due to loss of follow-up. Kaplan-Meier results highlighted that ccRCC patients having up-regulated PFKFB3 expression experienced lower survival odds (Fig. 1f). Moreover, multivariate Cox analysis suggested PFKFB3 as a unique and potential prognostic biomarker for ccRCC (Table 1).

**Table 1:** Correlation between the PFKFB3 level and the overall survival of ccRCC patients

Clinical Variables	HR	95% CI	P-value
Univariate analysis			
Age (≥60 vs <60)	1.875	1.152-3.149	0.015
Gender (Male vs Female)	1.267	0.589-2.627	0.365
TNM stage (III/IV vsI/II)	3.396	2.133-5.548	<0.001
Furhman grade (III/IV vsI/II)	2.376	1.116-4.385	0.003
PFKFB3 (High vs Low)	1.715	1.232-2.540	<0.001
Multivariate analysis			
Age (≥60 vs <60)	1.394	0.925-2.214	0.065
TNM stage (III/IV vsI/II)	2.674	1.878-3.794	<0.001*
Furhman grade (III/IV vsI/II)	1.751	1.130-2.812	0.009*
PFKFB3 (High vs Low)	1.358	1.028-2.791	<0.001*

### Up-regulation of PFKFB3 expression in RCC cells

**Fig. 2:**
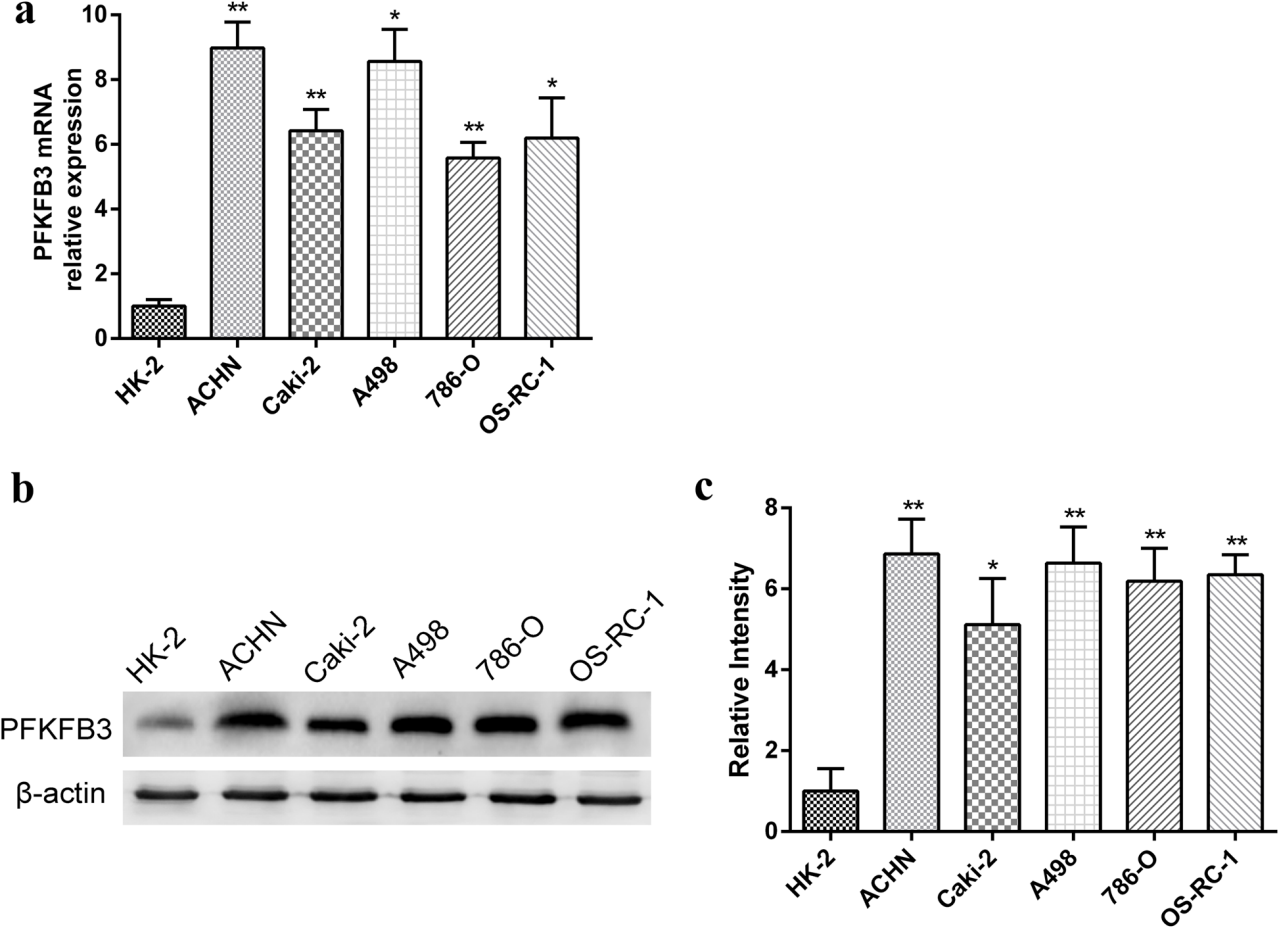
PFKFB3 is up-regulated in RCC. (**a**) PFKFB3 mRNA levels in a panel of RCC cell lines (ACHN, Caki-2, A498, 786-O and OS-RC-1) and healthy human proximal tubule epithelial cell line (HK-2), analyzed by qRT-PCR. (**b**) Protein levels for PFKFB3 in RCC cells were determined by Western blot analysis. (**c**) Protein levels of PFKFB3 were relatively quantified as the samples derive from the same experiment and that blots were processed in parallel. The results are presented as the mean ± SD. *, P < 0.05; **, P < 0.01

In order to evaluate the influence of PFKFB3 on RCC progression, this study initially employed RT-qPCR and Western blot to detect transcriptomic and proteomic PFKFB3 levels in an immortalized, normal human-proximal-tubule-epithelial cell line (HK-2), together with a panel of RCC cell lines (ACHN, Caki-2, A498, 786–0, OS-RC-1). Figure 2a depicted that the mRNA up-regulated PFKFB3 expression occurred in RCC cell lines, when comparing to HK-2 cells. Furthermore, Western blot results indicated that PFKFB3 protein expression levels were exacerbated in RCC cells, when compared with HK-2 cells, with statistical significance (Fig. 2b-c).

### Knockdown of PFKFB3 inhibits glycolysis in RCC cells

**Fig. 3:**
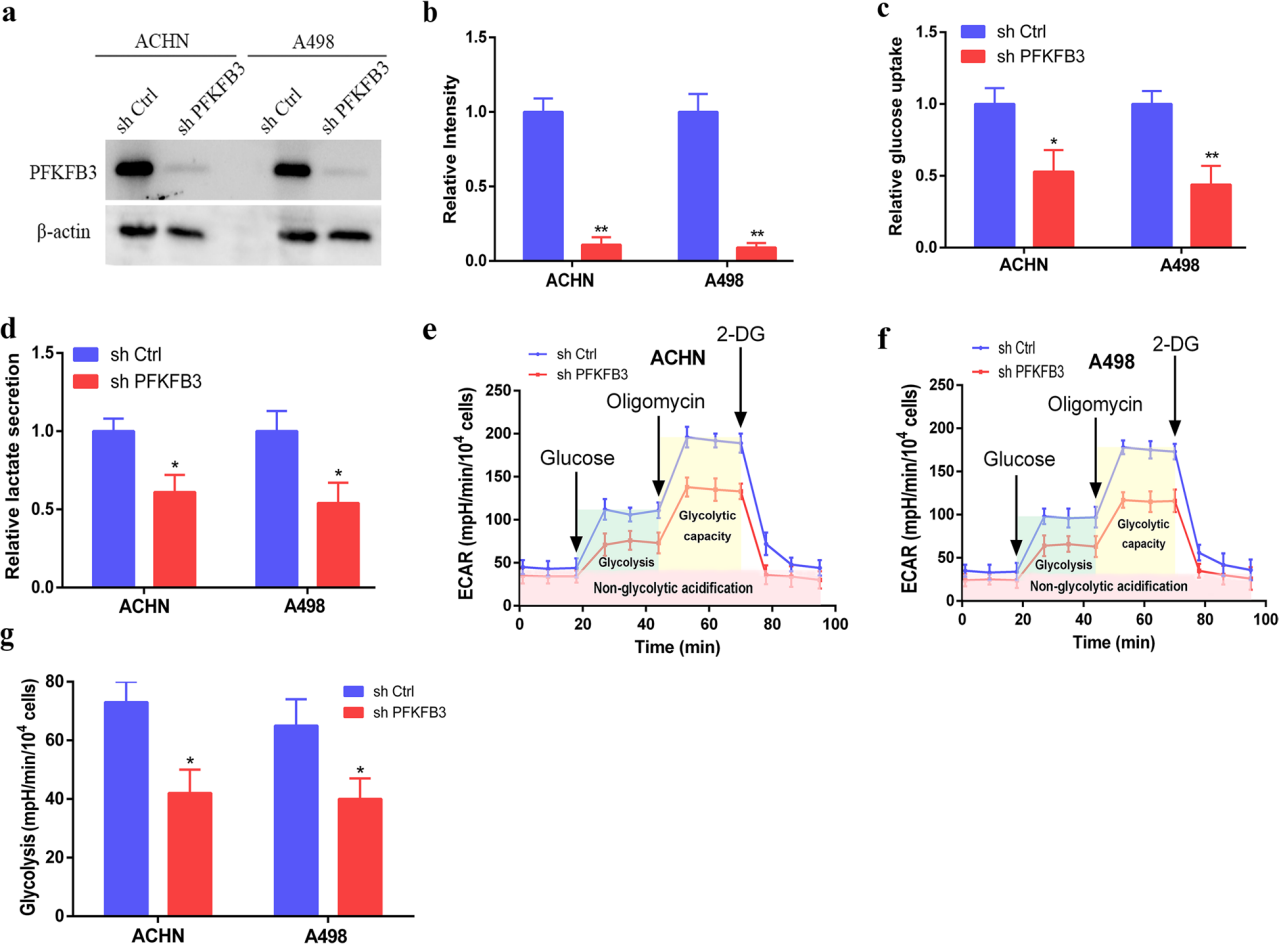
Knockdown of PFKFB3 inhibits glycolysis in RCC cells. (**a**) After shRNA transfection, Western blotting was employed as a validation measure for PFKFB3 knockdown in ACHN and A498 cell lines. (**b**) Protein levels of PFKFB3 were relatively quantified as the samples derive from the same experiment and that blots were processed in parallel. (**c-d**) Measurement of relative glucose uptake and lactate secretion in transfected cell lines. (**e-f**) ECAR levels were detected in transfected ACHN and A498 cells using an XF Extracellular Flux Analyzer. (**g**) Glycolysis (the ECAR increase following the addition of glucose) was further quantified by ECAR analysis. The results are presented as the mean ± SD. *, P < 0.05; **, P < 0.01

Considering PFKFB3 is a key protein that regulates cell glycolysis, this study further explored the effects of PFKFB3 knockdown on glycolysis in RCC cells. In this study, loss-of-function assays through PFKFB3 shRNA were carried out. ACHN and A498 cells were selected since such cell lines exhibited highest PFKFB3 transcriptomic-expression levels from all RCC cell line types (Fig. 2a). The knockdown efficiency for PFKFB3 was confirmed by Western blot (Fig. 3a-b). Figure 3c-d depicted that knockdown of PFKFB3 in ACHN and A498 cells strikingly reduced the glucose intake and lactate secretion capacity. Moreover, since the extracellular acidification rate (ECAR) for RCC was determined through the Seahorse XF24e® Extracellular Flux Analyzer, basal glycolysis, maximal glycolytic capacity and glycolytic reserve of ACHN and A498 cells were found to be restrained after transfection of PFKFB3 shRNA (Fig. 3e-g), which insinuated that glycolytic potency of RCC cells might be attenuated by PFKFB3 shRNA. In summary, such results suggest PFKFB3 to have major functional roles in RCC-cell aerobic glycolysis pathways.

### Down-regulation of PFKFB3 suppresses proliferation and G1/S transition in RCC cells

**Fig. 4:**
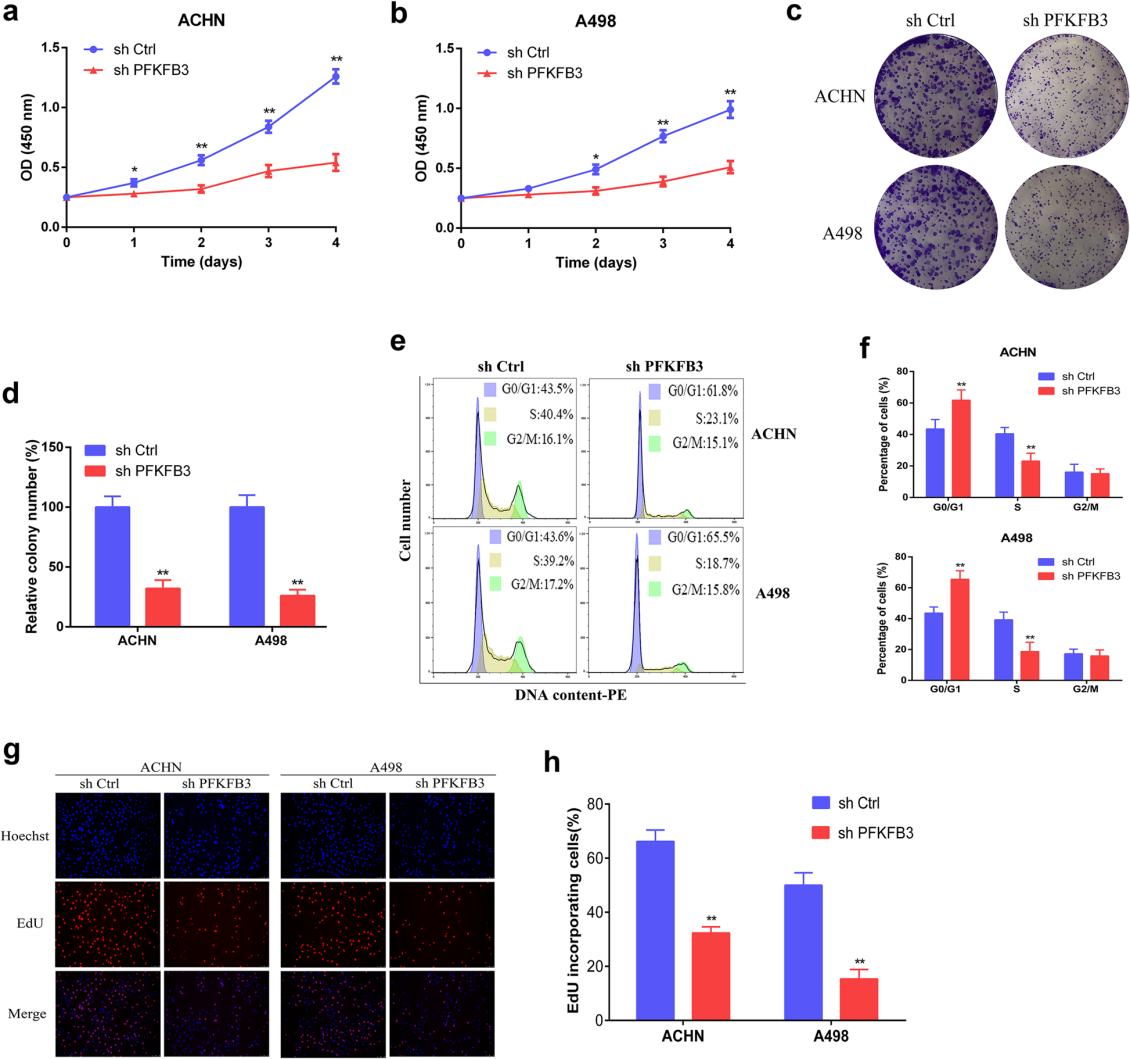
Downregulation of PFKFB3 suppresses proliferation and G1/S shifts within RCC. (**a**) CCK-8 investigations were employed for calculating cell viability. (**b-c**) Images / relative quantification of crystal violet-stained cell colonies identified through colony formation assays. (**d**) Flow cytometry assays for cell cycle distribution analysis. (**e**) Cell cycle phase investigation-based statistics. (**f-g**) Micrograph imaging and quantification of EdU-incorporated cells. The results are presented as the mean ± SD. *, P < 0.05; **, P < 0.01

CCK-8 assays demonstrated that PFKFB3 knockdown reduced cell proliferation ability of ACHN and A498 cell lines (Fig. 4a-b). Moreover, the colony formation assay confirmed both ACHN and A498 cells successfully formed fewer and smaller colonies after transfection by PFKFB3 shRNA (Fig. 4c-d). Figure 4e-f depicted that knockdown of PFKFB3 in ACHN and A498 cells led to significant rise of G1-phase-bound cells, together with lower cellular levels in S-phase. In addition, EdU incorporation assay demonstrated that ACHN and A498 cells contained reduced EdU-positive cells with novel synthesized DNA following transfection of PFKFB3 shRNA (Fig. 4g-h). Consequently, such data suggests that PFKFB3 might accelerate G1/S cell-cycle progressive shift, leading to exacerbated proliferative activity in RCC cells.

### PFKFB3 inhibitor 3PO suppresses glycolysis and growth in RCC cells

3-(3-Pyridinyl)-1-(4-pyridinyl)-2-propen-1-one (3-PO) has been widely reported as a novel compound that reduce glycolytic flux through competitive inhibition of PFKFB3 [15]. In the present study, cells were cultured in the medium added 3PO (10 μM) as the experimental group, and the control group was absence of 3PO. After examining glycolysis flux (glucose uptake and lactate secretion analysis) and cell viability (CCK8 assay), we found that 3PO significantly inhibited the glycolytic activity and proliferation of RCC cells (Fig. S1).

### PFKFB3 knockdown inhibits RCC tumor growth in the nude murine model

**Fig. 5:**
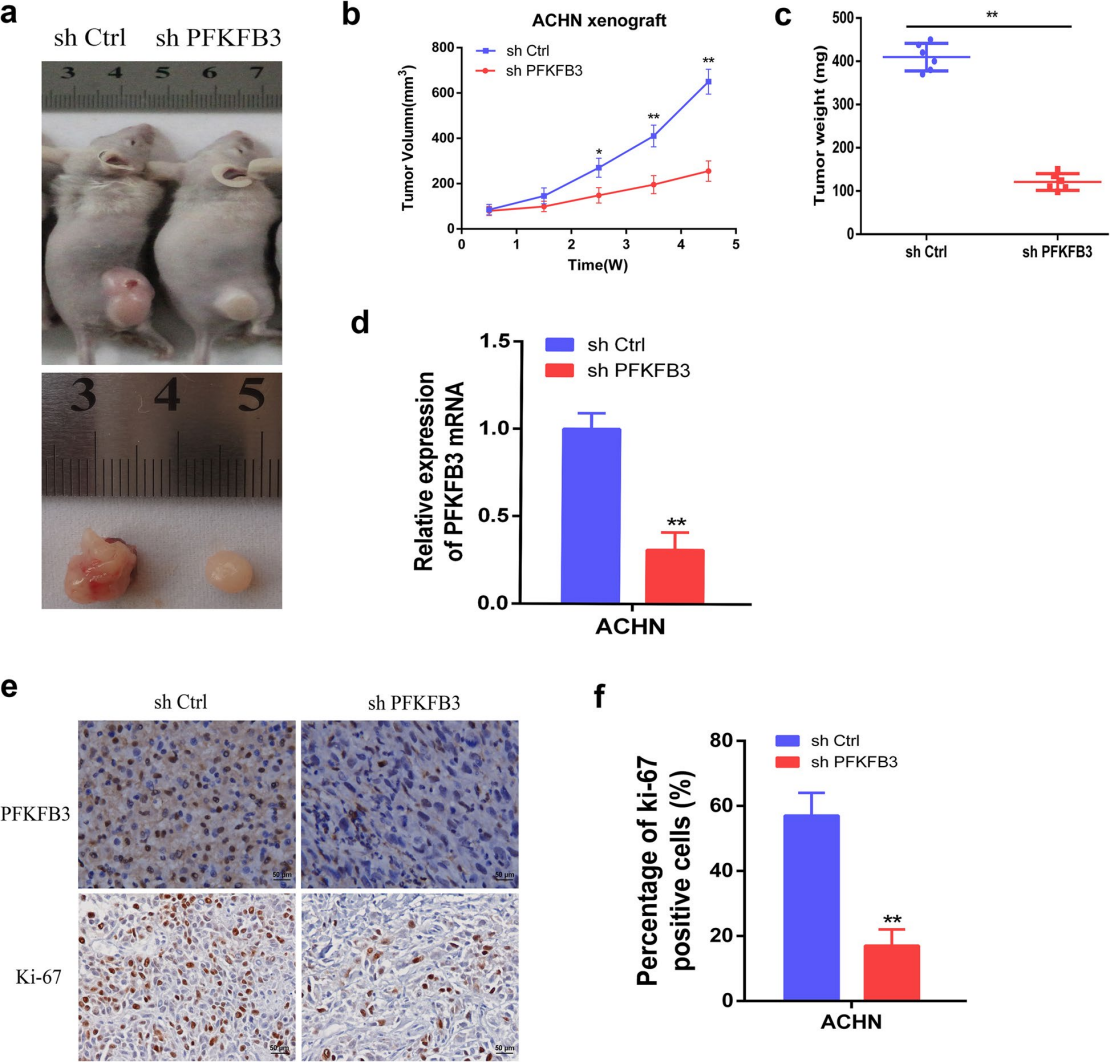
PFKFB3 knockdown inhibits RCC tumor growth in murine models. (**a**) Imaging of ACHN cell-based cancer formation in murines under different conditions. (**b**) Statistics for ACHN cell-based tumor volumes in nude murines (*n* = 6 per group). (**c**) Statistics for ACHN cell-based tumor weight in nude murines (n = 6 per group). (**d**) PFKFB3 mRNA levels were detected in tumors from different groups. (**e**) IHC imaging of PFKFB3 and Ki-67 in randomized ACHN cancer cells. (**f**) Quantification of IHC staining of Ki-67 in ACHN cell-based tumors. The results are presented as the mean ± SD. *, P < 0.05; **, P < 0.01

To test the impact of PFKFB3 on the growth of RCC tumor in vivo, ACHN cells with PFKFB3 shRNA were injected into nude mice tumors subcutaneously. As expected, tumors with PFKFB3 knockdown displayed a marked reduction in tumor weight/volume in comparison to the control arm of the study (Fig. 5a-c). Furthermore, qRT-PCR analysis revealed that PFKFB3 mRNA levels were significantly reduced in tumors with PFKFB3 knockdown (Fig. 5d). In addition, immunohistochemical staining confirmed that PFKFB3 knockdown successfully down-regulated Ki-67, an established cell proliferation biomarker (Fig. 5e-f). All such findings are in line with the in vitro results of this comprehensive investigation and, in summary, suggest that PFKFB3 down-regulation successfully impairs RCC xenograft tumor growth.

## Discussion

Tumors re-wire glucose metabolic processes to favor aerobic glycolysis, in order to exacerbate tumor size and continue un-regulated cell proliferative activity. PFKFB3 functions as a master controller on elevated glycolytic flow in tumors through synthesizing fructose 2,6-biphosphate, the latter in turn activating 6-phosphofructo-1-kinase. As a vital regulator of glycolysis, accumulating studies have reported that PFKFB3 is associated with many aspects of cancer, including carcinogenesis, cancer cell proliferation, vessel aggressiveness, drug resistance and tumor microenviroment [19]. Moreover, the pharmacological inhibition of glycolysis has emerged as a novel strategy for treating cancer since high glycolytic activity is considered as a metabolic hallmark of cancer, PFKFB3 has been also considered as a potential target in cancer treatment [20]. Multiple investigations also suggested that PFKFB3 is up-regulated in a spectrum of tumor models [9–11], while there are few reports on the expression of PFKFB3 in RCC. Our investigation analyzed PFKFB3 levels within 90 ccRCC patients, revealing up-regulated PFKFB3 expression in ccRCC. Moreover, ccRCC-based PFKFB3 overexpression was positively associated with tumor stage though independent of tumor grade, suggesting that PFKFB3 played pivotal parts in bridging glycolysis processes to cell proliferative rate changes in such tumor cells.

The majority of scientific literature stated that enhancing PFKFB3 exacerbates tumorigenesis and cell proliferative rates [20–23]. The study carried out on astrocytoma cells by Zscharnack et al. [24] gave contradicting results. This study revealed that PFKFB3 splice variant UBI2K4 was down-regulated within high-grade astrocytoma, in comparison to low-grade astrocytoma and non-neoplastic brain tissue. Construct-induced expression of UBI2K4 regulated cell viability and anchorage-independent growth by U87 cells. Following from this, more studies for identifying PFKFB3 roles affecting differing tumor models should be carried out. In the present study, endogenous PFKFB3 expression in all analyzed RCC cell lines was strikingly elevated in comparison to healthy human-proximal-tubule-epithelial cell line HK-2. Since there existed extremely elevated innate expression levels of PFKFB3 in the selected RCC cell types, loss-of-function experiments in vitro */* in vivo assays were carried out through PFKFB3 silencing for investigating the biological effect of PFKFB3. Selected RCC cell lines (ACHN and A498) were those having the most elevated innate expression levels for PFKFB3. Our loss-of-function studies demonstrated that PFKFB3 inhibition significantly decreased glycolysis and cell proliferative activities in RCC, both in vitro and in vivo.

Moreover, we found that PFKFB3 knockdown blocked the G1/S shift in RCC cells, indicating that such an impairment leads to reduced cell proliferative activity. One particular investigation reported reduced glycolytic activity following PFKFB3 gene silencing, together with leading to induction of G2 phase cell cycle arrest within HeLa cell cultures [25]. Even though the functions of PFKFB3 in glycolysis was well-studied, other researchers shifted focus onto other functional roles adopted by this gene. For example, Yalcin et al. [13] found that PFKFB3 knockdown triggered G1 phase cell cycle arrest and also up-regulated p27 in HeLa cell cultures, with p27 co-siRNA silencing leading to a reversal of PFKFB3 siRNA-induced silencing activity, mainly since p27 is a powerful regulator for G1/S shifts and is also pro-apoptotic in nature [26]. Other studies have also shown that PFKFB3 was transported to the nucleus in cancer cells, with external-influenced PFKFB3 wild-type expression within the nucleus led to exacerbated cell proliferative activity, without any influences on glycolytic processes [27]. This indicates that PFKFB3 functions in tumorigenesis are also due to its cell cycle regulatory effects, not just on the glucose metabolic pathway regulatory roles. The detailed relationship between these phenomena and the specific molecular mechanism of PFKFB3 still needed to be further explored.

This study did have its limitations. None of the RCC cell lines in this study had low basal endogenous PFKFB3 levels, consequently eliminating the prospect of performing gain-of-function analyses. Additionally, the exact molecular mechanism of PFKFB3 on the proliferation of RCC cells remains elusive.

## Conclusions

In essence, PFKFB3 is typically highly expressed in RCC, with over-expression of this gene being intimately correlated with late TNM stage and low prognostic odds in such ccRCC cases. PFKFB3 knockdown suppresses glycolysis, proliferation, and blocks the G1/S conversion in RCC cells. The results from this investigation indicate that PFKFB3 retains pivotal parts in RCC development and is an attractive drug target in RCC.

## Supplementary Information

**Additional file 1: Supplementary Fig. 1.** PFKFB3 inhibitor 3PO suppresses glycolysis and growth in RCC cells. (a-b) Measurement of relative glucose uptake and lactate secretion in ACHN and A498 cells treated with 3PO (10 μM). (c-d) CCK-8 investigations were employed for calculating cell viability in ACHN and A498 cells treated with 3PO (10 μM).

## Data Availability

The datasets used and/or analyzed during the current study are available from the corresponding author on reasonable request.

## Abbreviations

RCC: renal cell carcinoma
ccRCC: clear cell renal cell carcinoma
PFKFB3: phosphofructo-2-kinase/fructose-2,6-biophosphatase 3
IHC: immunohistochemistry
TMA: tissue microarray
EACR: extracellular acidification rate
3PO: 3-(3-Pyridinyl)-1-(4-pyridinyl)-2-propen-1-one
