# Supplementary figures and images for: Overexpression of PFKFB3 promotes cell glycolysis and proliferation in renal cell carcinoma
Source: BMC Cancer. 2022 Jan 20;22:83. doi: 10.1186/s12885-022-09183-2 (PMC8772232; doi:10.1186/s12885-022-09183-2)

**Original image for Figure 2b**


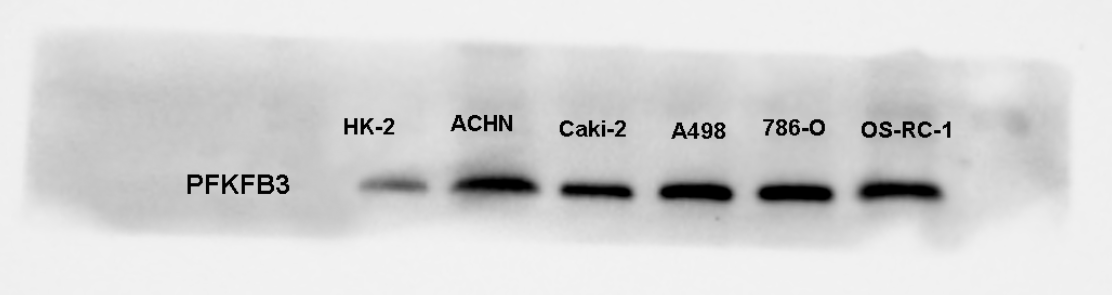


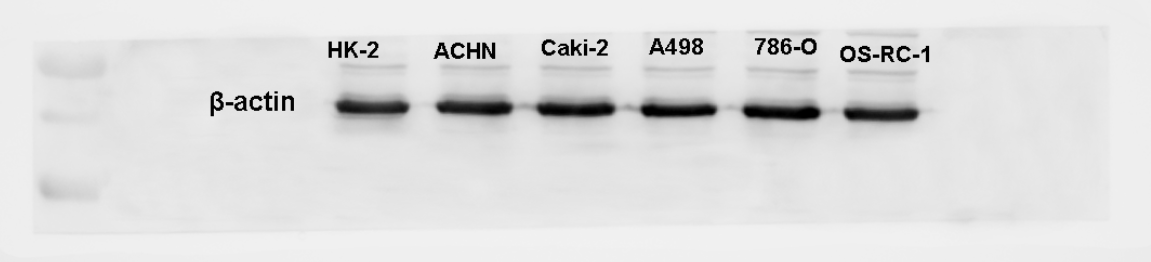


**Original image for Figure 3a**
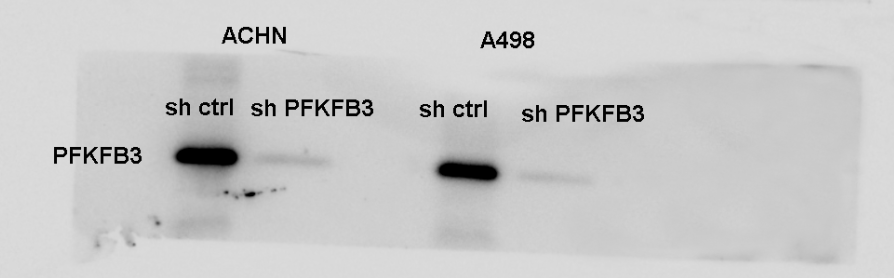

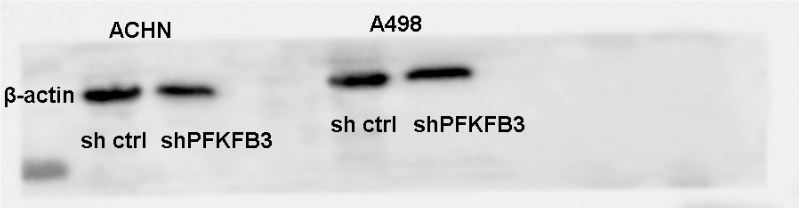

Supplement: Supplementary file 1 — Additional file 1: Supplementary Fig. 1. PFKFB3 inhibitor 3PO suppresses glycolysis and growth in RCC cells. (a-b) Measurement of relative glucose uptake and lactate secretion in ACHN and A498 cells treated with 3PO (10 μM). (c-d) CCK-8 investigations were employed for calculating cell viability in ACHN and A498 cells treated with 3PO (10 μM). [file 12885_2022_9183_MOESM1_ESM.zip › Original image of WB(12-5).docx]

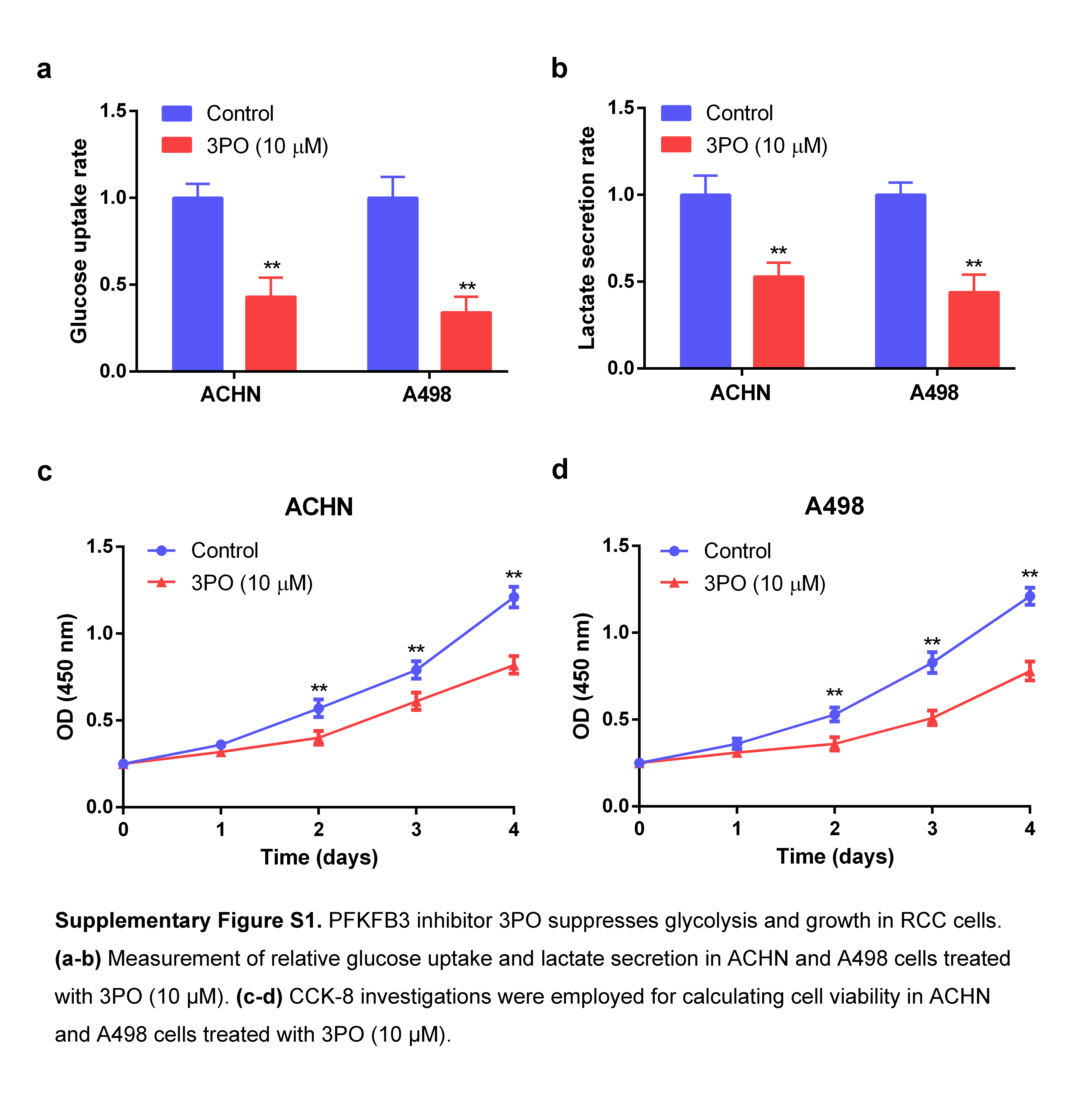

Supplement: Supplementary file 1 — Additional file 1: Supplementary Fig. 1. PFKFB3 inhibitor 3PO suppresses glycolysis and growth in RCC cells. (a-b) Measurement of relative glucose uptake and lactate secretion in ACHN and A498 cells treated with 3PO (10 μM). (c-d) CCK-8 investigations were employed for calculating cell viability in ACHN and A498 cells treated with 3PO (10 μM). [file 12885_2022_9183_MOESM1_ESM.zip › Supplementary Figure S1.tif]
